# Thinking Outside Malaria: A Rare Case of Disseminated Cysticercosis With Cardiopulmonary Involvement From Urban Tanzania

**DOI:** 10.7759/cureus.12851

**Published:** 2021-01-22

**Authors:** Frederick R Lyimo, Ahmed M Jusabani, Hilda Makungu, Maria Mtolera, Salim Surani

**Affiliations:** 1 Radiology, Muhimbili National Hospital, Dar es Salaam, TZA; 2 Radiology, Aga Khan Hospital, Dar es Salaam, TZA; 3 Internal Medicine, Corpus Christi Medical Center, Corpus Christi, USA; 4 Internal Medicine, University of North Texas, Dallas, USA

**Keywords:** cysticercosis, taenia solium, cardiopulmonary cysticercosis, seizure, epilepsy, brain mass

## Abstract

Dissemination of the cysticerci throughout the body with cardiopulmonary involvement represents a very rare occurrence and an uncommon form of cysticercosis manifestation. We report a rare case of a 48-year-old African male from urban Tanzania who was, at first, referred to our radiology department for a coronary computed tomography angiography (CCTA), but incidentally on further evaluation of the patient revealed a history of recurrent convulsions, loss of consciousness, a single episode of temporary loss of vision and recent skin nodules. The value of a full clinical and radiological evaluation of the patient presenting with adult-onset seizures cannot be overemphasized for the diagnosis of this disease. Management of disseminated cysticercosis is complex and, therefore, should be tailored to fit the individual cases and focus on clinical manifestations.

## Introduction

Cysticercosis is a parasitic infection resulting from cysticercus cellulose, a larval stage of the parasite *Taenia solium*. Tapeworm carriers transmit *T. solium *eggs to humans through the fecal-oral route [1,2].

About 50-100 million people globally are estimated to be infected by cysticercosis; however, this is considered an underestimate since many infections are silent, and there is a paucity of data on the actual prevalence [3]. A high prevalence of cysticercosis has been observed in most developing countries, especially in Latin-America, Sub-Saharan Africa, India, and Asia. This can be attributed to close contact between pigs and humans in these countries, with the superimposition of poor sanitary conditions [4,5].

Tanzania has a very high risk of cysticercosis owing to a growth in the piggery industry, leading to an increase in pork consumption in rural and urban areas [6]. Few available studies done on human cysticercosis have been based on neurocysticercosis and have reported a prevalence of 16.2% among people being investigated for epilepsy in Northern Tanzania [6].

About 16.7% of villagers from the southern highland region of Tanzania who participated in a cross-sectional survey tested positive for a cysticerci enzyme-linked immunosorbent assay antigen test (Ag-ELISA), while 45.3% were found to have anti-cysticercal antibodies. Among those positive for Ag-ELISA, 5.2% had positive computed tomography (CT) scan findings suggestive of neurocysticercosis [7]. 

Neurocysticercosis is the most common form of cysticercosis affecting the central nervous system (CNS) and a major cause of adult-onset seizures. Disseminated cysticercosis with cardiopulmonary involvement represents a rare manifestation of this disease, with less than 50 cases being reported worldwide by the year 2012 [1,2,8-12] and none from East Africa.

This case presentation aims to shed light on this rare form of disseminated cysticercosis, the complexity and challenges related to its diagnosis and management in a resource-poor setting like Tanzania.

## Case presentation

A 48-year-old African male was referred to our imaging department for a coronary computed tomography angiography (CCTA) due to a recent history of left side chest pain. Further evaluation of the patient revealed complaints of recurrent loss of consciousness for six years, convulsions for four years, a single episode of temporary loss of vision three years ago, and recent skin nodules. He is a known hypertensive patient diagnosed four years ago and is on regular medication with a fair response. He denied any known allergy or surgical history. There is no known history of tuberculosis contact or a known family history of epileptic disorders. He admitted to eating pork meat up to four times a week. He was a truck driver and is married with seven living children. This illness led him to stop driving. His wife and children are all doing well.

The first episode of loss of consciousness occurred suddenly and lasted for a few minutes. He denied having a prior illness. He was taken to a nearby hospital where he was treated for malaria and soon resumed normal activities.

He was doing well thereafter until the following year when he developed a second episode of a sudden loss of consciousness and woke up in a hospital two days later. During hospitalization, he was put on IV fluids and antimalarial drugs. This episode was followed by a severe headache that lasted for two days, temporarily relieved by acetaminophen tablets. Just like in the first episode, he had no prior illness.

In the third year from the onset of the first symptom, he developed an episode of generalized tonic-clonic convulsions associated with tongue biting, frothing from the mouth, and neck stiffness. This was followed by loss of consciousness. During hospitalization, he was once more treated for malaria.

**Figure 1:**
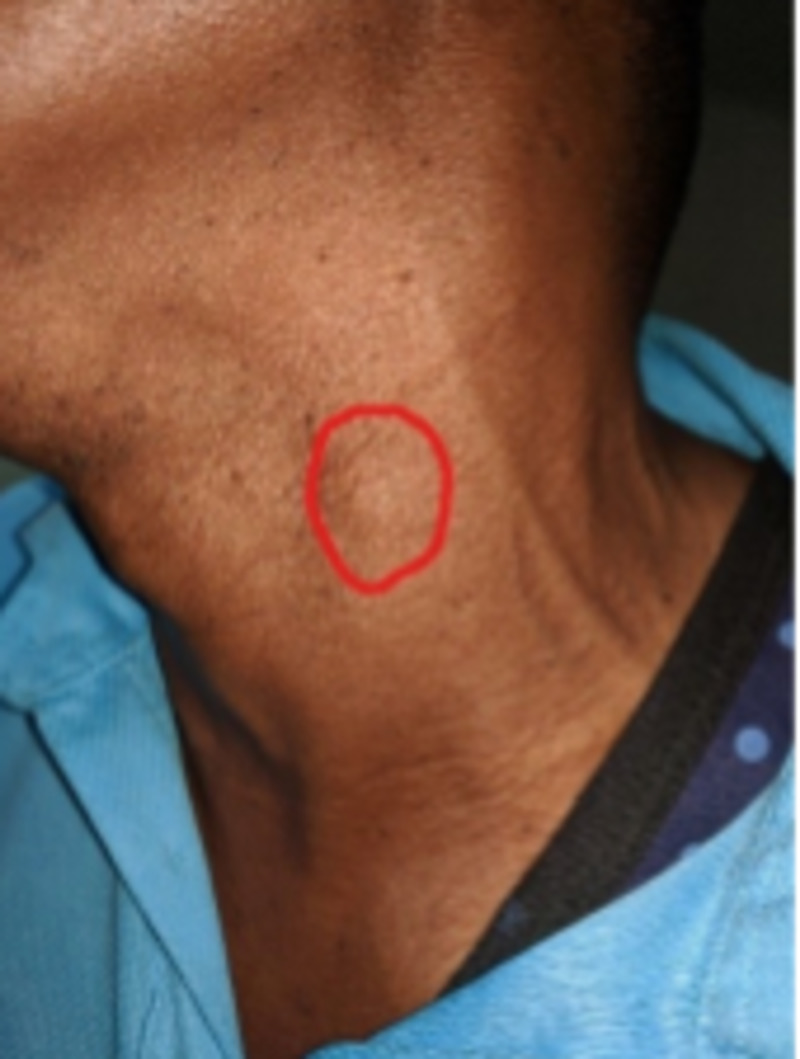
Photograph showing a subcutaneous skin nodule on the left side of the neck, which is circled red.

These episodes of convulsions and loss of consciousness kept recurring yearly with increasing frequency and the duration of loss of consciousness in each episode until when he reported at our facility. In the past few months prior to presentation, he discovered having small skin nodules distributed on the neck, chest, and back. They were immobile, painless, and had no associated skin color changes (Figure 1).

Physical examination revealed a stable adult patient, alert, afebrile, not pale or jaundiced, and with no lower limb edema. His pulse rate was 80 beats/min. Blood pressure was 141/90 mmHg. The respiratory exam revealed 28 breaths per minute, oxygen saturation on room air was 99%, and bronchovesicular breath sounds were heard. Normal S1 and S2 sounds were heard on the cardiac exam. There was no murmur or thrill. Skin exam revealed few visible scattered palpable skin nodules at the neck, chest, and back. The rest of the systemic exam was unremarkable. A provisional diagnosis of disseminated cysticercosis and hypertensive heart disease was made. 

Hematological tests revealed a normal range of hemoglobin, leukocytes, and platelets. His blood lipid panel, blood renal, and liver profiles were unremarkable. Sickling test, rapid plasma reagin test for syphilis, and ELISA for the human immunodeficiency virus were all negative. Electrocardiography (ECG) exam revealed a normal sinus rhythm.

**Figure 2:**
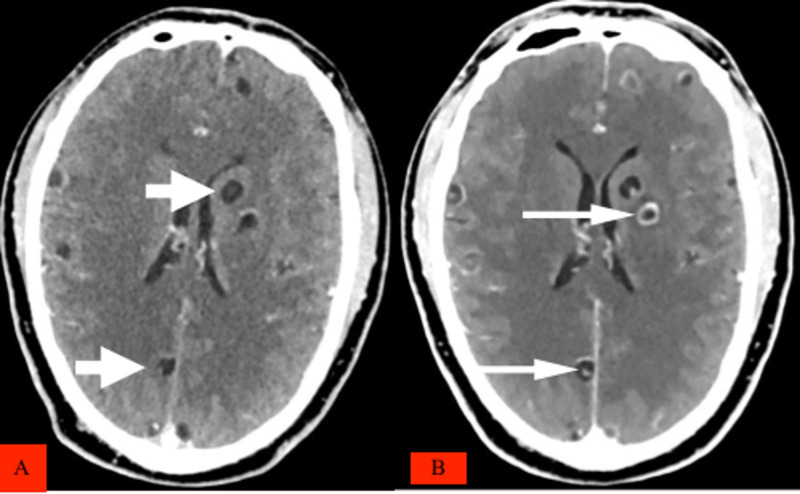
Axial IV contrasted brain CT showing (A) numerous cystic hypodense lesions pointed by short arrows, and (B) fewer calcified cystic lesion six weeks after treatment, some with a dot sign pointed by long arrows.

CT scan of head and neck, chest, abdomen, and pelvis revealed multiple cystic lesions with dot sign diffusely distributed in the brain, neck, chest, including the heart, abdomen, and pelvis (Figure 2).

**Figure 3:**
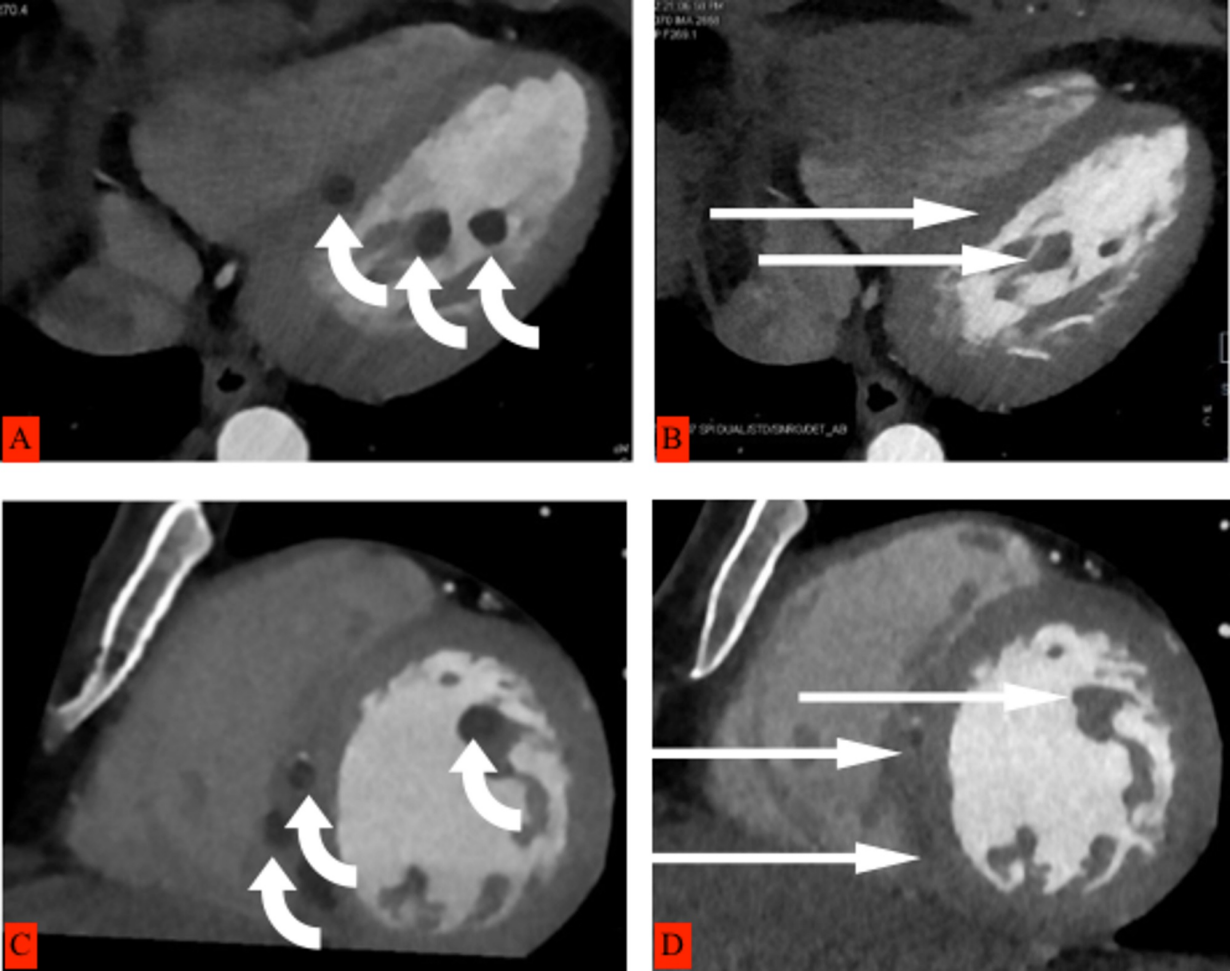
Cardiac CT images on the left side (A, C) showing multiple hypodense cystic lesions pointed with curved arrows at the interventricular septum and papillary muscles before treatment, and on the right side (B, D), significantly smaller and fainter lesions pointed with long arrows six weeks after treatment.

**Figure 4:**
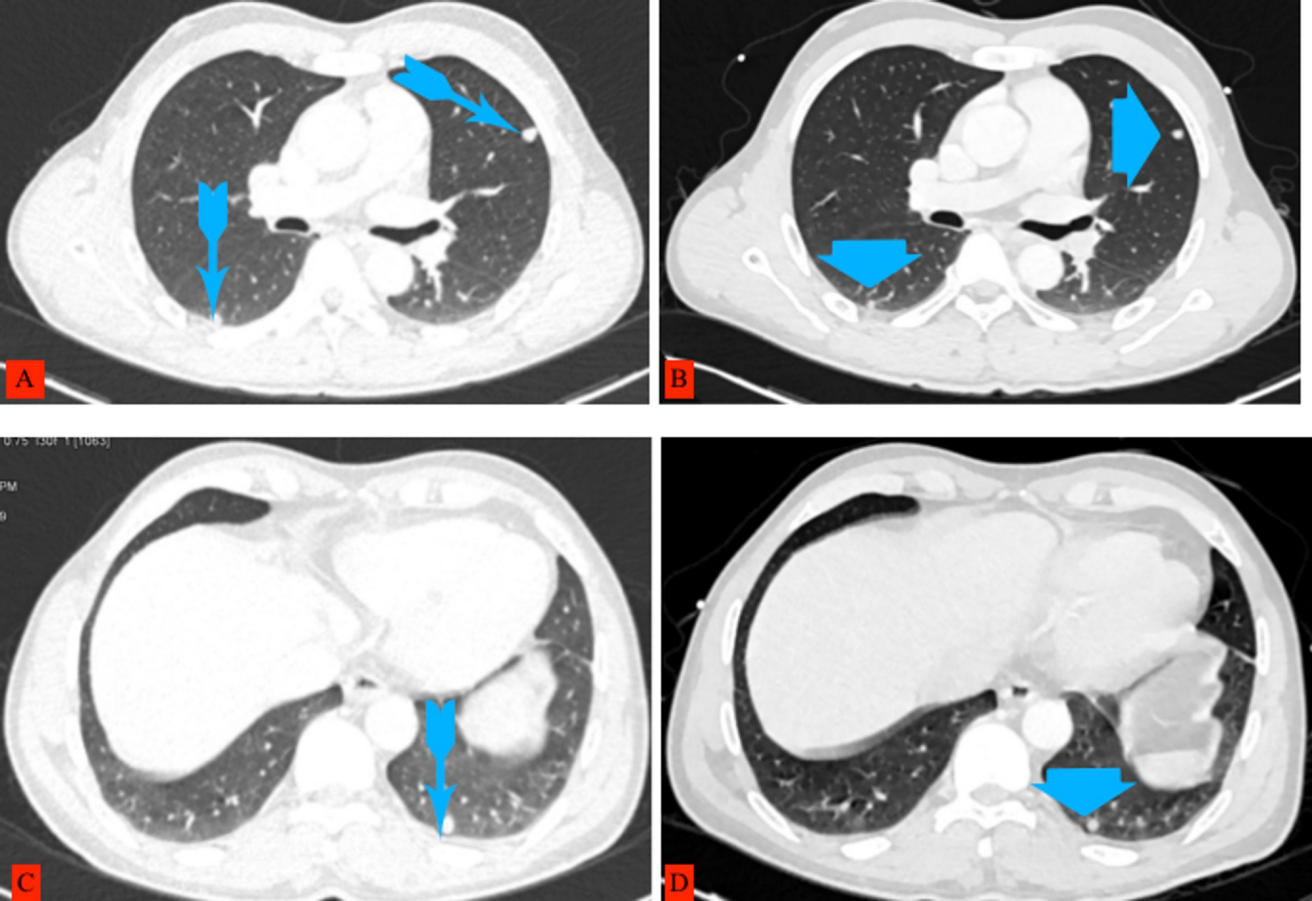
Chest CT images on the left side (A, C) showing nodular lesions pointed with arrows with tails before treatment, and on the right side (B, D), the same lesions pointed with arrowheads but reduced in size six weeks after treatment.

**Figure 5:**
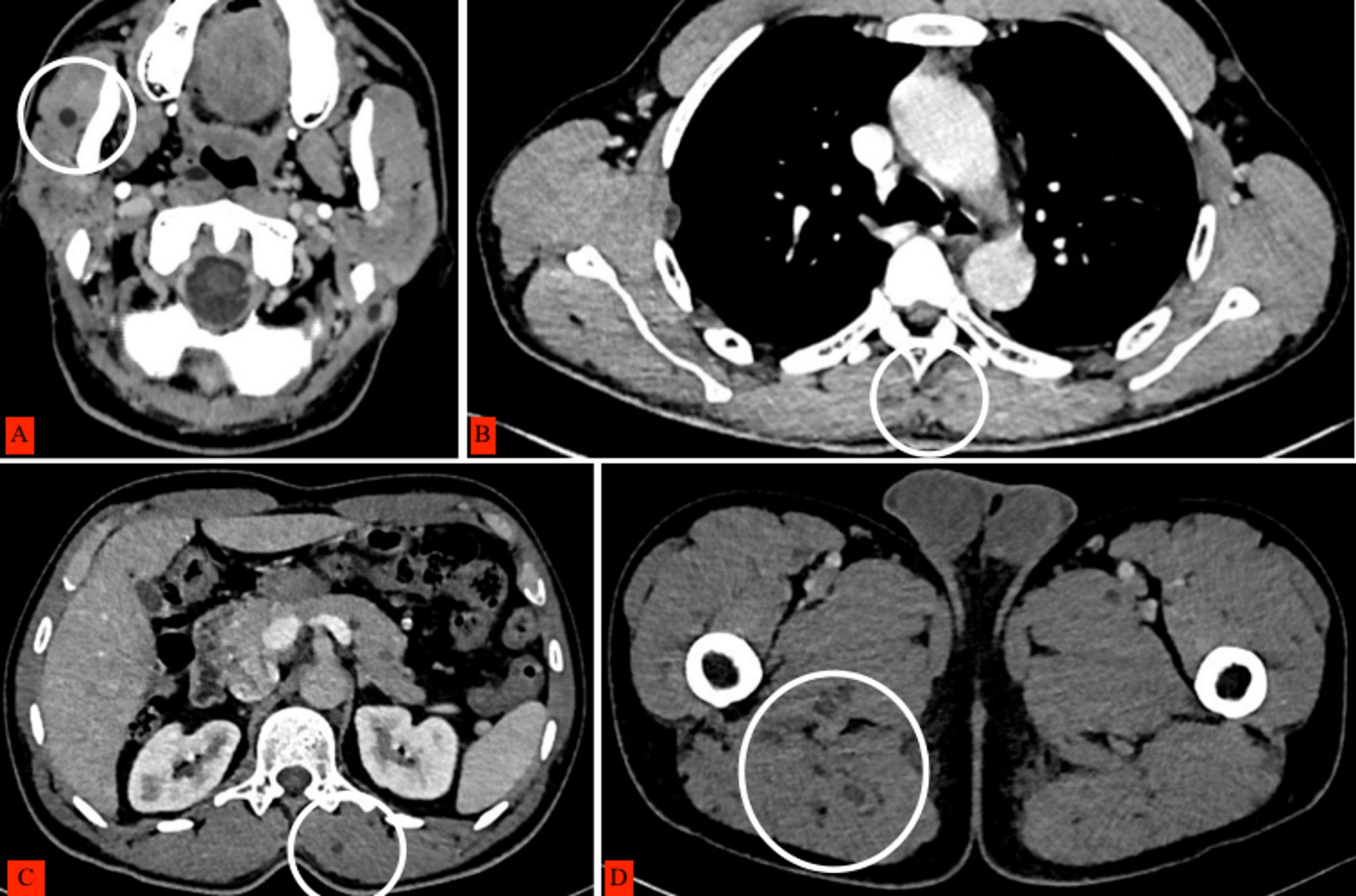
Axial IV contrasted images showing cysticerci as hypodense lesions involving the (A) head, (B) chest, (C) abdomen, and (D) pelvic muscles, which have been circled.

Cardiac CT images showed multiple hypodense cystic lesions at the interventricular septum and papillary muscles (Figure 3). CT chest, abdomen, and pelvis showed cystic lesions (Figures 4, 5). Excision biopsy of two subcutaneous nodules was performed and confirmed cutaneous cysticercosis on the pathological exam. A final diagnosis of disseminated cysticercosis with cardiopulmonary involvement was made based on imaging and histology.

The patient was treated with albendazole 400mg twice a day for two weeks, followed by 400mg once a day for two weeks, prednisone 40mg, which was tapered down, and carbamazepine 200mg twice a day for two weeks. During treatment, the patient did not complain of seizures or loss of consciousness or headache. A follow-up CT scan confirmed that the cysts had markedly reduced in number and size, and many had become non-viable (Figures 2B, 3B, 3D, 4B, 4D). The patient is still on albendazole 400mg once a day and is regularly seen in the outpatient clinic with no further seizures with the plan to continue the albendazole for another four weeks.

## Discussion

Humans acquire cysticercosis through ingestion of *T. solium* eggs contained in the feces from human tapeworm carriers. Transmission can occur directly from a carrier or indirectly from eating food prepared by the carrier, thus explaining why vegetarians may acquire the disease. Although it is uncommon, auto-infection is also known to occur. Eating undercooked cysticerci infected pork does not cause cysticercosis but rather taeniasis (adult tapeworm intestinal infection) and a *T. solium* eggs carrier state [13,14].

Oncospheres released from ingested eggs penetrate the intestinal mucosa and get disseminated via the hepatoportal system to different tissues and body organs [15]. The most common sites these larval cysts are found include the central nervous system, skeletal muscle, subcutaneous tissue, the eyes, and occasionally the heart [9,15].

Clinical manifestations of cysticercosis are related to the immune status of the patient, the location and numbers of cysticerci in the organ or tissue affected depending on its size, consistency, and its blood supply.

Cardiac and pulmonary involvement of cysticercosis was previously thought to be extremely rare. Reasons for the rarity may be explained by the fact that adult larvas prefer brain and skeletal muscles to complete their life cycle. A second reason would be their asymptomatic nature since patients usually present with CNS symptoms. Autopsy studies have shown cardiac involvement in 20%-25% of patients with documented CNS cysticercosis. Patients with cysticercosis with cardiac involvement may present with arrhythmias and conduction abnormalities [10,12]. Our patient neither had arrhythmia symptoms nor abnormal ECG findings.

A computed tomography scan is an invaluable tool that can diagnose cysticercosis with high sensitivity and specificity. A number of lesions, anatomical location, natural history, and stages of treatment can be demonstrated [1,2,16]. There is a marked similarity of cysticercosis findings in all the affected organs. Usually, for these cases, there are innumerable thin-walled cystic appearing lesions that are isodense with cerebrospinal fluid (CSF) and have a hyperdense “dot” within them, representing a scolex. Although not pathognomonic, identification of a scolex highly suggests the cysticercosis diagnosis [10,17]. 

In pulmonary cysticercosis, a CT scan usually shows multiple random nodules of varying sizes. These cannot be distinguished from other causes, including metastatic disease [10]. Our patient’s CT scan showed multiple hypodense lesions, some with a dot sign diffusely distributed in all organs, including the heart muscles and a few pulmonary nodules. This was sufficient to suggest cardiopulmonary pulmonary involvement of the disseminated cysticercosis along with the improvement with albendazole therapy.

A follow-up CT scan for monitoring the progress of our patient after four weeks revealed fewer and smaller lesions; some had calcified while others demonstrated enhancing walls without perilesional edema, suggesting non-viable cysticerci as demonstrated by others [2].

Magnetic resonance imaging (MRI) is also a very useful imaging modality in disseminated neurocysticercosis for diagnosis, anatomical localization of the cysts, and documenting the natural history of the disease. MRI is more sensitive than CT as it has advantages in identifying scolex and live cysts, cysts in the CSF spaces in the brain and spine, and monitoring the response of the disease to therapy [18].

Treatment of disseminated cysticercosis is rather complex and must consider multiple factors such as symptomatology, location, size, and stage of cysts. Patients with high infection burden may require a longer course of treatment with anti-parasitic medication. Specific anthelminthic therapy with albendazole and praziquantel has proven to be highly efficacious against cysticercosis. Symptomatic treatment of CNS lesions with corticosteroids and anticonvulsants may be required in disseminated cysticercosis [2,5,14,19].

To the best of our knowledge, this is the first case of this nature to be documented in East Africa as per literature search in PubMed and Google Scholar. Even though we do not have an established guideline for the treatment of disseminated cysticercosis in Tanzania, our patient responded very well to the given regimen.

## Conclusions

Cysticercosis is a tropical disease that is an increasing healthcare burden in both urban and rural communities. The parasite can manifest in any organ of the human body causing direct or indirect damage with or without clinical symptoms. Having a high index of suspicion for this disease and the value of complete clinical and radiological evaluation of patients presenting with adult-onset seizures cannot be emphasized enough. In malaria-endemic areas, other differentials for seizures must be given due consideration, especially in populations where the pig is regularly consumed. Diagnosis and management of disseminated cysticercosis are complex and, therefore, should be tailored to fit the individual case and focus on clinical manifestations.
